# Supplementary material for: Content-rich biological network constructed by mining PubMed abstracts
Source: BMC Bioinformatics. 2004 Oct 8;5:147. doi: 10.1186/1471-2105-5-147 (PMC528731; doi:10.1186/1471-2105-5-147)
Supplement: Additional File 2 — The original results of the above study (non-essential files are deleted to keep the file size under the limit set by BMC bioinformatics). [file 1471-2105-5-147-S2.bz2 › chilibotAdditionalFile2/dip10/298ID7486683E1289/html/PIK3R1_PDGFRB.html]

 


 **PIK3R1** and **PDGFRB** 
  
Found 21 abstracts in PubMed, retrieved 10.  
 

 What does Google say? 
 PDF only 
| .edu only 

---

**Interactive relationship** (e.g. stimulation, inhibition, etc)

**Inhibitory relationship**- We describe strategies to i target EGFR, its ligand independent variant EGFRvIII, and PDGFR  [ **PDGFRB** ]  on the cell surface, ii inhibit constitutively activate RAS MAPK and PI3K  [ **PIK3R1** ]  Akt signaling pathways, iii target TP53 mutant tumors, and iv block GBM angiogenesis and invasion.  Ref: 12580545 Brain Pathol, 2003
**Stimulatory relationship**- However, binding of a diphosphotyrosine containing peptide that mimics an activated platelet derived growth factor receptor beta  [ **PDGFRB** ]  induced an increase in the apparent molecular mass of PI3K  [ **PIK3R1** ] .  Ref: 9837914 J Biol Chem, 1998
**Neutral relationship**- Most likely this was due to the presence of a suramin insensitive intracellular PDGFR  [ **PDGFRB** ]  pool that allowed activation of PI3K  [ **PIK3R1** ]  located in intracellular compartments.  Ref: 11473364 J Cell Physiol, 2001

**Non-interactive relationship** (e.g. studied together, co-existance, homology, etc.)

- In conclusion, our results demonstrate that Gi enables the PDGFR  [ **PDGFRB** ]  to signal more efficiently to p42 p44 MAPK, and this appears to be achieved through the regulation of c Src and Grb  [ **PIK3R1** ]  2 PI3K  [ **PIK3R1** ] , which are intermediates in the p42 p44 MAPK cascade.  Ref: 9882612 Biochem J, 1999
- To investigate the in vivo relevance of this observation, intracellular inositol trisphosphate IP3 generation and calcium release were examined in HepG2 cells expressing a series of PDGFR  [ **PDGFRB** ]  mutants that activate PLCgamma with or without receptor association with PI3K  [ **PIK3R1** ] .  Ref: 9726983 J Biol Chem, 1998
- Moreover, we further demonstrated a requirement for the activation of phosphatidylinositol 3 kinase PI3K  [ **PIK3R1** ]  in this response by using chemical inhibitors of PI3K  [ **PIK3R1** ] , mutant PDGFR  [ **PDGFRB** ] , and dominant negative PI3K  [ **PIK3R1** ] .  Ref: 10197615 Cancer Res, 1999
